# Risk factors for postpartum depression: an umbrella review

**DOI:** 10.3389/fpubh.2025.1714668

**Published:** 2026-01-22

**Authors:** Min Xu, Yuting Luo, Yuehua Huang, Yunxia Liu, Lingling Ding

**Affiliations:** Obstetrics and Gynecology Department, The First Affiliated Hospital of Sun Yat-Sen University, Guangzhou, China

**Keywords:** evidence, postpartum depression, preventive strategies, risk factors, umbrella review

## Abstract

**Objective:**

This umbrella review aimed to systematically evaluate and synthesize the evidence on risk factors associated with postpartum depression (PPD), assess the methodological quality and credibility of the existing meta-analyses, and identify high-priority targets for prevention and intervention.

**Methods:**

We systematically searched PubMed, Embase, Web of Science, Cochrane Library, and Medicine up to March 2025 for systematic reviews and meta-analyses reporting on risk factors for PPD. Eligible studies were appraised using AMSTAR-2 and GRADE frameworks. Risk factors were categorized based on effect estimates, heterogeneity, publication bias, and evidence strength.

**Results:**

Seventy-seven meta-analyses were included, covering 53 unique risk factors. Among these, 40 showed statistically significant associations with PPD. Unintended pregnancy (OR: 1.55), intimate partner violence (OR: 2.50), poor social support (RR: 3.57), sleep disorders (OR: 2.36), and history of depression (OR: 3.09) emerged as the strongest risk factors. Protective factors included postpartum physical activity (SMD: −0.42), doula support (RR: 0.36), breastfeeding support (MD: −2.11), and parenting interventions (SMD: −0.34). Most evidence was rated as low or very low in certainty; only a few outcomes based on randomized controlled trials were graded as moderate to high quality.

**Conclusions:**

PPD is influenced by a broad spectrum of psychosocial, biological, and obstetric risk factors. Although many associations are supported by statistical significance, the overall evidence quality remains suboptimal. Targeted screening and preventive strategies should prioritize high-risk groups, while future research should focus on high-quality, prospective studies to establish causal links and improve maternal mental health outcomes.

**Systematic review registration:**

PROSPERO (Registration No.: CRD420251249033).

## 1 Introduction

Postpartum depression (PPD) is a prevalent and debilitating mental health condition that affects a significant proportion of new mothers globally. Characterized by persistent sadness, fatigue, anxiety, and impaired bonding with the infant, PPD can have profound effects not only on the wellbeing of the mother but also on the infant's development and the overall family dynamic. Global estimates suggest that ~10%−20% of women experience PPD, with rates even higher in low- and middle-income countries (1). Despite being recognized as a major public health concern, the etiology of PPD remains multifactorial and poorly understood, involving a complex interplay of biological, psychological, and social factors. Various systematic reviews and meta-analyses have explored these risk factors, but their heterogeneity in methods and conclusions poses challenges for clinicians and policymakers aiming to implement effective screening and preventive strategies (2, 3).

To address this issue, umbrella reviews—an approach that synthesizes and grades evidence from existing systematic reviews and meta-analyses—have emerged as a powerful method to provide high-level summaries. Unlike traditional reviews, umbrella reviews systematically evaluate the quality, consistency, and strength of evidence across multiple reviews, allowing for more definitive conclusions regarding causality and public health relevance (4). This method is particularly valuable in the field of maternal mental health, where risk factors for PPD range from demographic and socioeconomic characteristics to biological and lifestyle-related exposures. For instance, unintended pregnancy (3), poor breastfeeding support (5), and vitamin D deficiency (6) have been implicated in elevating PPD risk, though the quality of supporting evidence varies widely. Consequently, there remains a pressing need for a rigorous synthesis of existing meta-analyses to identify which risk factors are most convincingly linked to PPD and warrant clinical attention.

This umbrella review aims to systematically assess and synthesize existing meta-analyses on the risk factors associated with postpartum depression. By categorizing the strength of associations using established evidence grading tools such as GRADE and AMSTAR, this study seeks to distinguish between well-established and weakly supported risk factors. The review also identifies gaps in the literature and provides recommendations for future research and clinical practice. Ultimately, our findings intend to guide healthcare providers, researchers, and policymakers in prioritizing effective screening strategies, tailoring interventions, and integrating mental health care into maternal health services to reduce the burden of PPD.

## 2 Methods and analysis

### 2.1 Design and registration

We adhered to PRISMA guidelines, (7) in systematically searching, extracting, and analyzing data from meta-analyses on postpartum depression risk factors. This umbrella review followed the Joanna Briggs Institute Manual for Evidence Synthesis (8) and the Cochrane Handbook for Systematic Reviews (9). This umbrella review was registered in PROSPERO (Registration No.: CRD420251249033).

### 2.2 Eligibility criteria

Meta-analyses on postpartum depression risk factors, encompassing all ethnicities, sexes, countries, and settings, were included. Data on individual risk factors were extracted separately when multiple factors were reported in a meta-analysis. The most recent meta-analysis was included if multiple analyses on the same risk factor were published more than 24 months apart. For studies conducted within 24 months, preference was given to those with the largest number of prospective cohorts, or, in the case of equal cohort numbers, the higher AMSTAR score (10). Both meta-analyses were included if one conducted a dose-response analysis and the other did not. Meta-analyses on postpartum depression mortality, as well as studies in non-English languages, animal models, non-dietary factors, and cell cultures, were excluded.

### 2.3 Population

This umbrella review evaluates meta-analyses on postpartum depression risk factors, emphasizing those that may elevate or reduce risk. Studies on mortality, as well as factors associated with exacerbation and recurrence, were excluded because these outcomes are not directly related to postpartum depression and do not contribute relevant information for identifying PPD-specific risk factors.

### 2.4 Exposure

At least one postpartum depression risk was identified, with assessments based on odds ratios (OR), relative risks (RR), hazard ratios (HR), and 95% confidence intervals (CIs).

### 2.5 Study designs

Only meta-analyses of studies on postpartum depression risk factors across diverse demographics and settings were eligible. These reviews focused on dietary risk factors and employed methods such as search strategy, inclusion/exclusion criteria, quality assessment, and data analysis. Original studies could include randomized controlled trials (RCTs), prospective or retrospective cohorts, case-control studies, or cross-sectional studies.

### 2.6 Information sources

This study searched PubMed, Embase, Web of Science, Medicine, and the Cochrane Database up to March 2025 for systematic reviews and meta-analyses on postpartum depression risk factors, and examined reference lists to identify additional articles.

### 2.7 Search strategy

In accordance with SIGN guidelines, databases were searched using MeSH terms, keywords, and text related to postpartum depression risk factors: (((((((Postpartum Period) OR (Period, Postpartum)) OR (Postpartum)) OR (Postpartum Women)) OR (Women, Postpartum)) OR (Puerperium)) AND ((((((Depression) OR (Depressive Symptoms)) OR (Depressive Symptom)) OR (Symptom, Depressive)) OR (Emotional Depression)) OR (Depression, Emotional))) AND ((meta-analysis) OR (systematic review)). The search strategy is detailed in Supplementary Table S1.

### 2.8 Study selection

**Figure 1:**
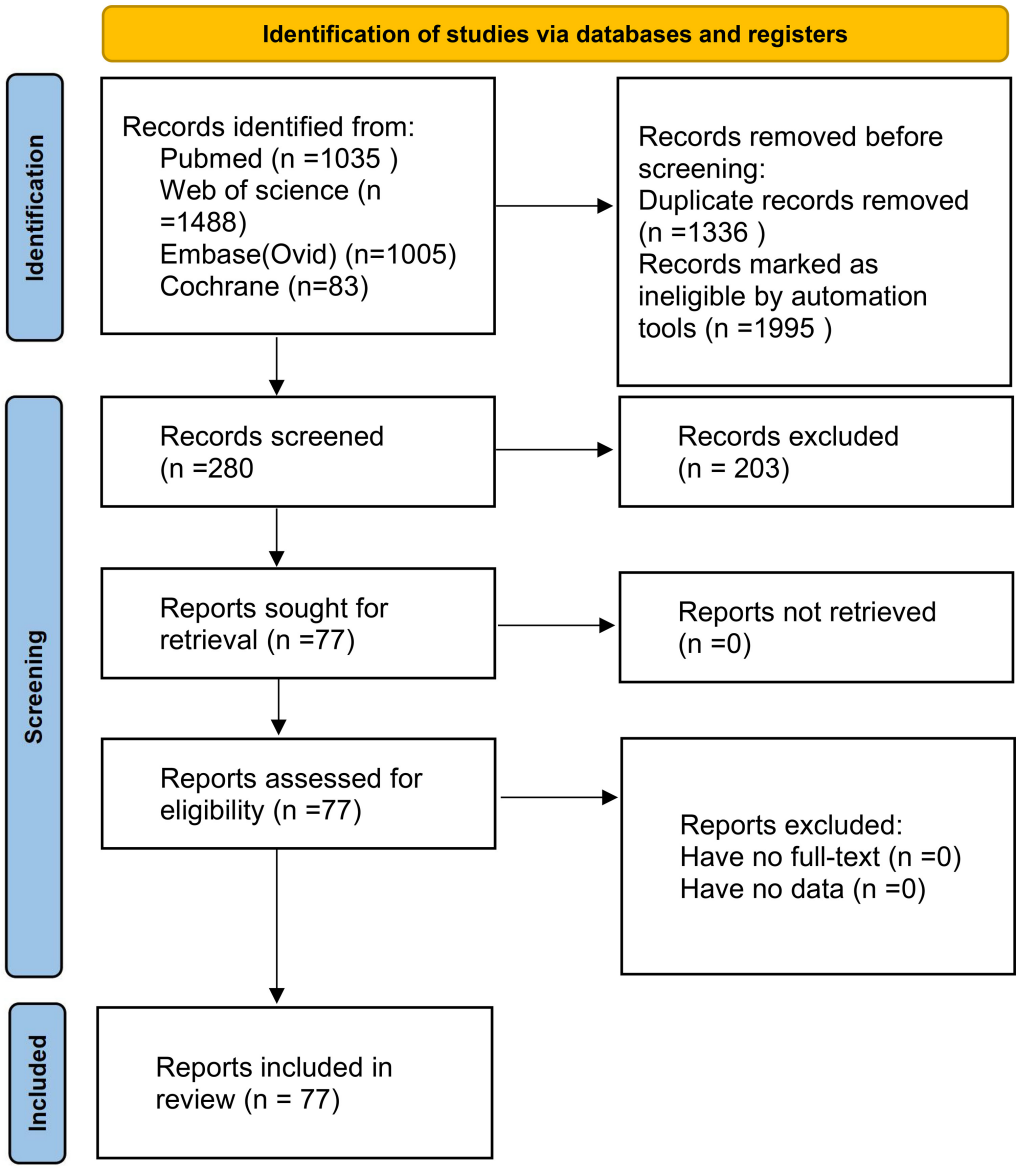
Flow chart.

Literature was screened in Endnote 20, and duplicates were removed. Two authors independently reviewed titles and abstracts to identify eligible meta-analyses, with discrepancies resolved by a third author. Reference lists were manually searched for additional meta-analyses (Figure 1).

When multiple meta-analyses addressed the same risk factor, we selected the most suitable analysis using prespecified criteria such as publication year and the number of prospective cohort studies included. When both dose–response and non–dose–response analyses were available, they were retained together only if they contributed complementary evidence. We also reviewed the primary studies within each meta-analysis to avoid repeated inclusion of identical original studies. These procedures reduced the risk of duplication bias and strengthened the reliability of the synthesized evidence.

### 2.9 Assessment of methodological quality

Two authors evaluated each meta-analysis using the AMSTAR tool (10, 11), and rated outcomes as “high,” “moderate,” “low,” or “very low” according to the GRADE system (12). Outcomes were categorized into five levels: Class I (convincing), Class II (highly suggestive), Class III (suggestive), Class IV (weak), and NS (nonsignificant) (10–12) (Table 1).

**Table 1:** Evidence categories criteria.

**Evidence class**	**Description**
Class I: convincing evidence	>1,000 cases (or >20,000 participants for continuous outcomes), statistical significance at *P* < 10^−6^ (random-effects), no evidence of small-study effects and excess significance bias; 95% prediction interval excluded the null, no large heterogeneity (*I*^2^ < 50%)
Class II: highly suggestive evidence	>1,000 cases (or >20,000 participants for continuous outcomes), statistical significance at *P* < 10^−6^ (random-effects) and largest study with 95% CI excluding the null value
Class III: suggestive evidence	>1,000 cases (or >20,000 participants for continuous outcomes) and statistical significance at *P* < 0.001
Class IV: weak evidence	The remaining significant associations with *P* < 0.05
NS: non-significant	*P* > 0.05

### 2.10 Data extraction

Two investigators independently extracted data from eligible studies, including author names, publication dates, risk factors, study sizes, case counts, total participants, study designs, and risk estimates (RR, OR, HR) with 95% CIs. Meta-analytic methods, heterogeneity (*I*^2^, Cochran's *Q*), and publication bias (Egger's, Begg's, funnel plot) were also recorded. For studies with dose-response or subgroup analyses, nonlinearity *P*-values and subgroup estimates were noted. Discrepancies were resolved by a third investigator.

### 2.11 Data summary

RR, OR, or HR with 95% CIs were recalculated using random or fixed effects models. Heterogeneity (*I*^2^, Cochran's *Q*) and small-study effects (Egger's or Begg's test for meta-analyses with >10 studies) were assessed when data allowed. For Class I or II evidence risk factors, sensitivity analyses evaluated the impact of individual studies on significance, provided data were sufficient. Dose-response analyses for postpartum depression risk factors were extracted from included meta-analyses. If the latest meta-analysis lacked clinical studies present in others, these were re-analyzed collectively. Heterogeneity was considered significant at *P* < 0.10, and other tests at *P* < 0.05. Evidence synthesis was performed in Review Manager v5.4.1, with Egger's, Begg's, and sensitivity analyses conducted in Stata v15.1. Risk factors were classified as significant or non-significant based on the statistical significance of the pooled effect estimates. Specifically, an association was considered significant when the 95% confidence interval did not cross the null value (1.0 for OR/RR and 0 for SMD/MD), and non-significant when the interval crossed the null line, regardless of whether the variable was binary or continuous.

## 3 Major outcomes

### 3.1 Characteristics of meta-analyses

The literature search and selection process is depicted in Figure 1. Following the search, 2,893 unique articles (i.e., records remaining after removing duplicates across all databases) were identified, which were then further screened by title/abstract and full text according to the predefined eligibility criteria, resulting in 77 meta-analyses (3, 5, 6, 13–27) that met our inclusion criteria. These meta-analyses synthesized evidence from multiple study designs, including randomized controlled trials, cohort studies, case-control studies, and cross-sectional studies. We extracted 53 risk factors, including 40 significantly and 13 non-significantly associated with postpartum depression (Table 2). Our analysis identified 37 adverse and 16 favorable associations with statistical significance. Thirty-nine outcomes were classified as Class IV (low quality) or NS (non-significant) after quality assessment. Only 14 risk factors achieved Class I and III evidence. Unplanned pregnancy (28) is classified as Class I evidence and is an important inducement that increases the risk of postpartum depression (Table 2).

**Table 2:** Risk factors of postpartum depression.

**Study**	**Risk factors**	**Assessed with**	**Total eligible MA**	**outcome**	**Included MA**	**No. of cases/total**	**MA metric**	**Estimates [95% CI]**	**No. of studies (*T*/*R*/*C*/*P*)**	**Effects model**	***I*^2^; *Q* test *P*-value**	**Egger test*P*-value**	**AMSTAR Scores**	**Evidence classification**	**Grade**
Nelson 2022 (28)	Unintended pregnancy	Unintended vs. intended pregnancy	3	Postpartum depression risk	Nelson 2022	1,000+/19,744+	OR	1.55 [1.38–2.03]	10/0/10/0	Random-effects	7.1%; 0.38	0.16	9	Class I	Very low
Adina 2022 (13)	Parenting interventions	With vs. without	1	score	Adina 2022	737/1,497	SMD	−0.34 [−0.44, −0.24]	15/15/0/0	Random-effects	0%; < 0.001	NA	9	Class IV	High
Adjie 2024 (5)	Breastfeeding support	BF intervention vs. standard care	3	EPDS score	Adjie 2024	1,280/2,516	MD	−2.11 [−3.18, −1.04]	10/10/0/0	Random-effects	92%; < 0.00001	0.031	9	Class III	Very low
Caffieri 2023 (27)	COVID-19	With vs. without	4	Postpartum depression risk	Caffieri 2023	54/NA	ES	0.26 [0.23, 0.30]	54/0/0/54	Random-effects	98.18%; 0	0.26	10	Class IV	Low
Wang 2018 (29)	25(OH)D level	< 50 vs. ≥50 nmol/L	5	Postpartum depression risk	Wang 2018	8,470	OR	3.67 [1.72, 7.85]	4/0/4/0	Random-effects	79.4%; 0.002	0.004	8	Class IV	Very low
Azami 2019 (22)	Gestational diabetes	NA	5	Postpartum depression risk	Azami 2019	2,370,958	RR	1.59 [1.22–2.07]	18/0/18/0	Random-effects	87.50%; 0.001	0.197	8	Class III	Very low
Falah-Hassani 2015 (30)	Immigrant women	Immigrant women vs. native women	2	Postpartum depression risk	Falah-Hassani 2015	33,047	OR	1.84 [1.32–2.57]	5/0/5/0	Random-effects	71.1%; 0.008	0.081	7	Class III	Very low
Wei 2024 (31)	Intimate partner violence	NA	5	Postpartum depression risk	Wei 2024	388,966	OR	2.5 [2.12, 2.95]	76/0/76/0	Random-effects	90%; NA	0.221	7	Class II	Very low
Li 2020 (32)	Five HTTLPR polymorphism	Patients vs. healthy	1	Postpartum depression risk	Li 2020	519/1,256	OR	0.54 [0.35, 0.82]	6/0/6/0	Random-effects	58%; 0.03	0.690	8	NS	Very low
Kang 2020 (33)	Anemia	NA	2	Postpartum depression risk	Kang 2020	32,792,378	OR/RR	1.53 [1.32–1.78]	7/0/7/0	Random-effects	81.4%; NA	0.7	8	Class II	Very low
Gong 2017 (34)	Doule delivery	Doule delivery vs. control	1	Postpartum depression risk	Gong 2017	82/1,513	RR	0.36 [0.29, 0.46]	13/13/0/0	Fixed-effects	0%; 0.80	NA	6	Class IV	Moderate
Ning 2024 (35)	Cesarean section	Cesarean section vs. natural vaginal delivery	4	Postpartum depression risk	Ning 2024	844,328	OR	1.12 [1.04–1.20]	8/0/8/0	Fixed-effects	47%; 0.06	0.82	9	Class IV	Low
Zeleke 2021 (36)	Poor social support	Poor vs. strong social support	1	Postpartum depression risk	Zeleke 2021	NA	OR	3.57 [2.29, 5.54]	3/0/0/3	Random-effects	53.8%; 0.115	NA	9	Class IV	Low
Li 2023a (37)	Sleep disorders	Sleep disorders vs. control	2	Postpartum depression risk	Li 2023a	NA/12,614	OR	2.36 [1.72, 2.32]	16/0/16/0	Random-effects	84%; < 0.00001	NA	9	Class IV	Very low
Cadman 2024 (26)	PM2.5	Single exposure	1	Postpartum depression risk	Cadman 2024	3,049/30,622	OR	1.04 [0.97, 1.11]	12/0/12/0	Random-effects	11.97%; NA	NA	5	NS	Very low
Cadman 2024 (26)	Road traffic noise	Lden ≥65 dB	2	Postpartum depression risk	Cadman 2024	23,956/2,346	OR	1.12 [0.95, 1.32]	11/0/11/0	Random-effects	12.95%; NA	NA	5	NS	Very low
Liu 2022 (38)	History of depression	With vs. without	2	Postpartum depression risk	Liu 2022	NA	OR	3.09 [1.62, 5.93]	4/0/4/0	Random-effects	86.5%; 0	0.309	9	Class III	Low
Cárdenas 2025 (39)	Maternal birth complacations	With vs. without	1	Postpartum depression risk	Cárdenas 2025	1,853,282	OR	1.47 [1.34, 1.61]	61/0/61/0	Random-effects	NA/ < 0.001	0.02	7	Class II	Very low
Chen 2019a (40)	Prenatal smoking	With vs. without	1	Postpartum depression risk	Chen 2019a	1,717/57,997	OR	2.32 [1.92, 2.81]	13/0/13/0	Random-effects	77.2%; 0	0.97	8	Class II	Very low
Chen 2019b (41)	Infertility treatment	With vs. without	1	Postpartum depression risk	Chen 2019b	NA	OR	1.21 [0.95, 1.56]	11/0/11/0	Random-effects	46.4; 0.045	NA	7	NS	Low
Chen 2019b (41)	Infertility treatment	With vs. without	1	Postpartum depression risk	Chen 2019b	NA	OR	0.89 [0.68, 1.16]	4/0/4/0	Random-effects	0; 0.49	NA	7	Class IV	Low
Chen 2019b (41)	Infertility treatment	With vs. without	1	Postpartum depression risk	Chen 2019b	NA	OR	0.56 [0.33, 0.96]	2/0/2/0	Random-effects	0; 0.78	NA	7	NS	Low
Cong 2021 (42)	Skin-to-skin contact	With vs. without	1	Score	Cong 2021	300/585	SMD	−0.72 [−1.08, −0.35]	6/6/0/0	Random-effects	75%; 0.001	NA	8	Class IV	Moderate
Molyneaux 2014 (43)	Obese women	Obese women vs. normal weight control	1	Postpartum depression risk	Molyneaux 2014	NA	OR	1.30 [1.20, 1.42]	15/0/NA/NA	Random-effects	49%; 0.014	NA	7	Class IV	Low
Davenport 2018 (63)	Prenatal exercise	With vs. without	2	Score	Davenport 2018	537/1,033	SMD	−0.01 [−0.13, 0.12]	4	Random-effects	0%; 1.00	NA	7	NS	Very low
Deprato 2024 (44)	Postpartum physical activity	With vs. without	1	Score	Deprato 2024	1,471/2,949	SMD	−0.42 [−0.62, −0.22]	35/35/0/0	Random-effects	83%; < 0.00001	NA	10	Class IV	Moderate
Ding 2023 (45)	Prenatal stressful life events	With vs. without	1	Postpartum depression risk	Ding 2023	9,822	OR	1.82 [1.52, 2.17]	17/0/17/0	Random-effects	66.9%; 0	0.503	9	Class IV	Very low
Eduardo 2019 (46)	Preterm birth	Preterm infants vs. full-term	2	Postpartum depression risk	Eduardo 2019	8,261	OR	1.79 [1.46, 2.21]	12/0/12/0	Fixed-effects	67%; 0.0006	NA	7	Class IV	Very low
Gallego-Gómez 2024 (47)	Urinary	With vs. without	1	Postpartum depression risk	Gallego-Gómez 2024	92,974	OR	1.45 [1.11, 1.79]	11/0/11/0	Random-effects	65.9%; 0	NA	8	Class IV	Very low
Ghanbari-Homaie 2024 (64)	Epidural analgesia	With vs. without	4	Postpartum depression risk	Ghanbari-Homaie 2024	1,004	OR	1.01 [0.64, 1.38]	3/0/3/0	Random-effects	77.65%; 0	0.04	7	NS	Very low
Zacher Kjeldse 2022 (48)	Family history of psychiatric disorders	With vs. without	1	Postpartum depression risk	Zacher Kjeldse 2022	103,458	OR	2.08 [1.67, 2.59]	25/0/25/0	Random-effects	57.14%; < 0.001	NA	10	Class IV	Very low
Ye 2020 (49)	Female	Female vs. male infant	2	Postpartum depression risk	Ye 2020	119,736	OR	1.15 [1.01, 1.31]	25/0/25/0	Random-effects	75%; < 0.00001	< 0.10	8	Class IV	Very low
Zhang 2019 (50)	Violence	With vs. without	3	Postpartum depression risk	Zhang 2019	177,531	OR	2.04 [1.72, 2.41]	32/0/26/6	Random-effects	93.7%; 0	0.709	7	Class IV	Very low
Wei 2025 (51)	Marital satisfaction	Low vs. high	1	Postpartum depression risk	Wei 2025	2,933	RR	3.47 [1.96, 6.12]	5/0/5/0	Random-effects	69.8%; 0.005	0.15	9	Class IV	Low
Wei 2025 (51)	Single/divorced/separated/widowed/unmarried	–	1	Postpartum depression risk	Wei 2025	70,563	RR	1.19 [1.02, 1.39]	11/0/11/0	Random-effects	52.8%; 0.007	0.22	9	Class IV	Very low
Tung 2022 (52)	winter	Winter vs. other seasons	1	Postpartum depression risk	Tung 2022	9,315/66,498	RR	0.89 [0.83, 0.96]	5/0/1/4	Random-effects	65%; < 0.00001	NA	8	Class IV	Very low
Zhao 2024 (53)	Assisted reproductive technologies	With vs. without	1	Postpartum depression risk	Zhao 2024	4,990/106,338	OR	0.83 [0.71, 0.97]	12/0/12/0	Random-effects	62.0%; 0.022	NA	6	Class IV	Very low
Schoretsanitis 2024 (54)	Postpartum hemorrhage	With vs. without	1	Postpartum depression risk	Schoretsanitis 2024	1,504/46,508	OR	1.28 [1.13, 1.44]	10/0/5/5	Random-effects	99%; < 0.01	0.23	9	Class III	Very low
Schoretsanitis 2022 (55)	Polycystic ovary syndrome	With vs. without	1	Postpartum depression risk	Schoretsanitis 2022	44,167/934,922	OR	1.45 [1.18, 1.79]	6/0/6/0	Random-effects	>50%; < 0.001	NA	9	Class III	Very low
Qiu 2022 (56)	Maternal alcohol	With vs. without	2	Postpartum depression risk	Qiu 2022	50,377	OR	1.21 [1.04, 1.41]	6/0/6/0	Random-effects	56%; 0.009	0.304	8	Class IV	Very low
Neda-Stepan 2024 (73)	Neuroticism	With vs. without	1	Postpartum depression risk	Neda-Stepan 2024	NA	OR	1.37 [1.22, 1.53]	13/0/13/0	Random-effects	88.3%; 0	< 0.001	9	Class III	Very low
Pacho 2023 (58)	Substance abuse	With vs. without	1	Postpartum depression risk	Pacho 2023	16,778/485,305	OR	3.67 [2.31, 5.85]	18/0/18/0	Random-effects	97%; < 0.01	NA	9	Class II	Low
Lu 2024 (59)	Postpartum pain	With vs. without	1	Postpartum depression risk	Lu 2024	3,973	OR	1.29 [1.10, 1.52]	8/0/8/0	Random-effects	66.5%; 0.004	0.009	9	Class IV	Very low
Minaldi 2020 (61)	Thyroid autoimmunity	TPOAb- vs. TPOAb+	1	Postpartum depression risk	Minaldi 2020	449/2,932	RR	1.49 [1.11–2.00]	5/0/5/0	Random-effects	47%; 0.11	NA	9	Class IV	Low
Mo 2022 (60)	Perinatal pain	With vs. without	1	Postpartum depression risk	Mo 2022	12,889/93,663	OR	1.43 [1.23, 1.67]	10/0/10/0	Random-effects	88.9%; 0	0.667	8	Class III	Very low
Li 2023b (65)	Neuraxial analgesia during labor	With vs. without	2	Postpartum depression risk	Li 2023b	767/43,803	RR	0.75 [0.56, 1.00]	13/0/13/0	Random-effects	79%; < 0.00001	NA	8	NS	Very low
Fu 2024 (62)	Adverse childhood experiences	With vs. without	2	Postpartum depression risk	Fu 2024	21,011	OR	2.31 [2.04, 2.63]	24/0/13/11	Random-effects	87%; < 0.01	< 0.05	9	Class IV	Very low
Cadman 2024 (26)	NO2	With vs. without	1	Postpartum depression risk	Cadman 2024	3,048/30,604	OR	0.98 [0.93, 1.03]	12/0/12/0	Random-effects	0; 0.295	NA	6	NS	Very low
Cadman 2024 (26)	PM2.5	With vs. without	1	Postpartum depression risk	Cadman 2024	3,049/30,622	OR	1.04 [0.97, 1.11]	12/0/12/0	Random-effects	0; 0.312	NA	6	NS	Very low
Cadman 2024 (26)	PM10	With vs. without	2	Postpartum depression risk	Cadman 2024	1,770/17,686	OR	1.08 [0.99, 1.17]	9/0/9/0	Random-effects	0; 0.723	NA	6	NS	Very low
Cadman 2024 (26)	NDVI	With vs. without	1	Postpartum depression risk	Cadman 2024	3,071/30,716	OR	0.99 [0.93, 1.05]	12/0/12/0	Random-effects	0%; 0.964	NA	6	NS	Very low
Cadman 2024 (26)	Green access	With vs. without	1	Postpartum depression risk	Cadman 2024	3,021/30,114	OR	0.98 [0.89, 1.07]	12/0/12/0	Random-effects	2%; 0.927	NA	6	NS	Very low
Cadman 2024 (26)	Blue access	With vs. without	1	Postpartum depression risk	Cadman 2024	4,006/31,099	OR	1.12 [1.00, 1.26]	12/0/12/0	Random-effects	0%; 0.94	NA	6	NS	Very low

### 3.2 Meta-anlysis results

In total, 53 risk factors were identified across the included meta-analyses, of which 40 showed statistically significant associations with postpartum depression and 13 were non-significant. These risk factors encompassed psychosocial, biological, obstetric, lifestyle, genetic, and environmental domains, reflecting the multifactorial nature of PPD etiology. Based on evidence class grading, 14 risk factors achieved Class I or Class II evidence, indicating comparatively stronger and more consistent associations across studies. The detailed findings for each significant and non-significant risk factor are summarized below.

#### 3.2.1 Significant risk factors

##### 3.2.1.1 Unintended pregnancy

**Figure 2:**
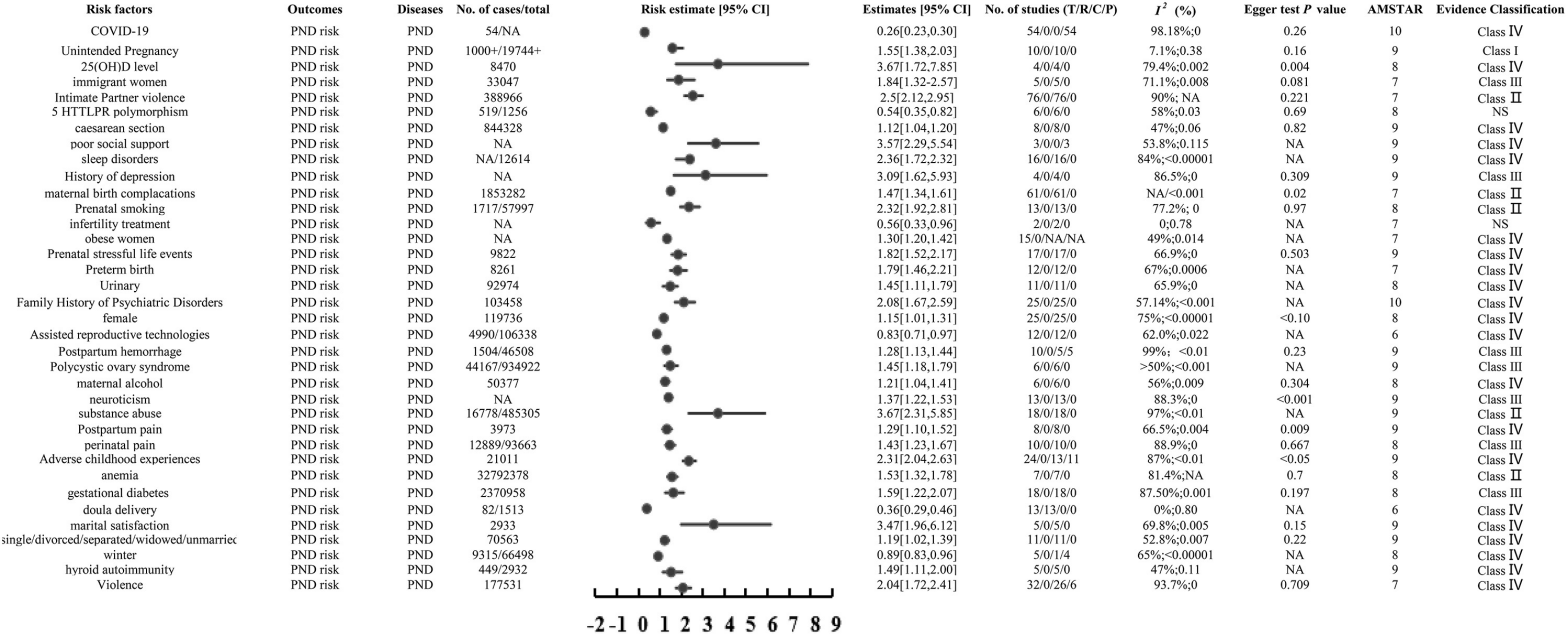
Forest plot of binary categorical variables.

A 2022 meta-analysis of 10 studies found that unintended pregnancy significantly reduces postpartum depression risk (OR: 1.55, 95% CI: 1.38, 2.03) (28) (Figure 2).

##### 3.2.1.2 Parenting interventions

A meta-analysis of 15 RCTs involving 737 cases and 1,497 participants found that parenting interventions significantly reduces the severity of postpartum depression (SMD: −0.34, 95% CI: −0.44, −0.24) (13) (Figure 2).

##### 3.2.1.3 Breastfeeding support

**Figure 3:**
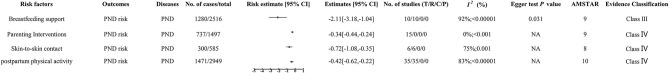
Forest plot of continuous variables.

Adjie's meta-analysis with 2,516 participants reported that breastfeeding support helps to alleviate postpartum depression and reduce its severity (MD: −2.11, 95% CI: −3.18, −1.04) (5) (Figure 3).

##### 3.2.1.4 COVID-19

A meta-analysis with 54 observational studies reported that during the COVID-19 pandemic, the risk of postpartum depression decreased instead (ES: 0.26, 95% CI: 0.23, 0.30) (27) (Figure 2).

##### 3.2.1.5 25(OH)D level

A 2018 meta-analysis with four cohort studies revealed that the lower the 25(OH)D level is, the higher the risk of postpartum depression (OR: 3.67, 95% CI: 1.72, 7.85) (29) (Figure 2).

##### 3.2.1.6 Gestational diabetes

A 2019 meta-analysis with 18 cohort studies showed that gestational diabetes can significantly increase the postpartum depression risk (OR: 1.59, 95% CI: 1.22, 2.07) (23) (Figure 2).

##### 3.2.1.7 Immigrant women

Falah-Hassani's meta-analysis with 33,047 participants reported that immigrant women have a higher risk of postpartum depression after childbirth (OR: 1.84, 95% CI: 1.32, 2.57) (30) (Figure 2).

##### 3.2.1.8 Intimate partner violence

Wei's meta-analysis with 388,966 participants reported that intimate partner violence is accompanied by a higher risk of postpartum depression (OR: 2.5, 95% CI: 2.12, 2.95) (31) (Figure 2).

##### 3.2.1.9 HTTLPR polymorphism

A 2020 meta-analysis with six cohort studies revealed that five HTTLPR polymorphism is accompanied by a lower risk of postpartum depression (OR: 0.54, 95% CI: 0.35, 0.82) (32) (Figure 2).

##### 3.2.1.10 Anemia

Kang's meta-analysis with 32,792,378 participants reported that women with anemia have a higher risk of postpartum depression (OR/RR: 1.53, 95% CI: 1.32, 1.78) (33) (Figure 2).

##### 3.2.1.11 Doula delivery

A meta-analysis with 13 RCTs reported that doula delivery can reduce the risk of postpartum depression (RR: .36, 95% CI: 0.29, 0.46) (34) (Figure 2).

##### 3.2.1.12 Cesarean section

A meta-analysis with 8 cohort studies reported that compared with the natural delivery method, the cesarean section has a higher risk of postpartum depression (RR: 1.12, 95% CI: 1.04, 1.20) (35) (Figure 2).

##### 3.2.1.13 Poor social support

Zeleke's meta-analysis with 3 studies reported that poor social support is accompanied by a higher risk of postpartum depression (RR: 3.57, 95% CI: 2.29, 5.54) (36) (Figure 2).

##### 3.2.1.14 Sleep disorders

A meta-analysis with 16 cohorts studies reported that sleep disorders are accompanied by a higher risk of postpartum depression (OR: 2.36, 95% CI: 1.72, 2.32) (37) (Figure 2).

##### 3.2.1.15 History of depression

A meta-analysis with four cohorts studies reported that pregnant women with a History of depression are accompanied by a higher risk of postpartum depression (OR: 3.09, 95% CI: 1.62, 5.93) (38) (Figure 2).

##### 3.2.1.16 Maternal birth complications

Cárdenas's meta-analysis with 1,853,282 participants reported that women with a maternal birth complications are more likely to have postpartum depression (RR: 1.47, 95% CI: 1.34, 1.61) (39) (Figure 2).

##### 3.2.1.17 Prenatal smoking

Chen's meta-analysis reported that prenatal smoking increases the risk of postpartum depression (OR: 2.32, 95% CI: 1.92, 2.81) (40) (Figure 2).

##### 3.2.1.18 Infertility treatment

A meta-analysis with two cohorts studies reported that Pregnant women with infertility treatment have a reduced risk of postpartum depression (OR: 0.56, 95% CI: 0.33, 0.96) (41) (Figure 2).

##### 3.2.1.19 Skin-to-skin contact

A meta-analysis with six RCTs showed that skin-to-skin contact can reduce the severity of postpartum depression (SMD: −0.72, 95% CI: −1.08, −0.35) (42) (Figure 3).

##### 3.2.1.20 Obese women

Molyneaux's meta-analysis with 15 studies depicted that compared with women of normal weight, obese women are more prone to postpartum depression (43) (OR: 1.30, 95% CI: 1.20, 1.42; Figure 2).

##### 3.2.1.21 Postpartum physical activity

A meta-analysis with 2,949 participants and 1,471 cases reported that postpartum physical activity reduces the degree of postpartum depression (SMD: −0.42, 95% CI: −0.62, −0.22) (44) (Figure 3).

##### 3.2.1.22 Prenatal stressful life events

Ding's meta-analysis with 17 cohorts studies depicted that experiencing stressful periods during pregnancy increases the risk of postpartum depression (OR: 1.82, 95% CI: 1.52, 2.17) (45) (Figure 2).

##### 3.2.1.23 Preterm birth

A meta-analysis with 8,261 participants reported that premature birth increases the risk of postpartum depression (OR: 1.79, 95% CI: 1.46, 2.21) (46) (Figure 2).

##### 3.2.1.24 Urinary

Gallego-Gómez's meta-analysis with 11 cohorts studies depicted that urinary increases the risk of postpartum depression (OR: 1.45, 95% CI: 1.11, 1.79) (47) (Figure 2).

##### 3.2.1.25 Family history of psychiatric disorders

A meta-analysis with 25 cohorts studies showed that having a Family History of Psychiatric Disorders will increase the risk of postpartum depression (OR: 2.08, 95% CI: 1.67, 2.59) (48) (Figure 2).

##### 3.2.1.26 Infant gender

Ye's meta-analysis with 25 cohorts studies depicted that when a baby girl is born, the risk of postpartum depression increases (OR: 1.15, 95% CI: 1.01, 1.31) (49) (Figure 2).

##### 3.2.1.27 Violence

A meta-analysis with 177,531 participants showed that Mothers who suffer from violence are more likely to develop postpartum depression (OR: 2.04, 95% CI: 1.72, 2.41) (50) (Figure 2).

##### 3.2.1.28 Marital satisfaction

Wei's meta-analysis with five cohorts studies depicted that mothers with low marital satisfaction are more likely to suffer from postpartum depression (RR: 3.47, 95% CI: 1.96, 6.12) (51) (Figure 2).

##### 3.2.1.29 Marital status

A meta-analysis with 70,563 participants showed that single/divorced/separated/widowed/unmarried people are more prone to postpartum depression (OR: 2.04, 95% CI: 1.72, 2.41) (51) (Figure 2).

##### 3.2.1.30 Childbirth season

Tung's study with 9,315 cases and 66,498 participants showed that babies are born in winter and mothers are less likely to suffer from postpartum depression (RR: 0.89, 95% CI: 0.83, 0.96) (52) (Figure 2).

##### 3.2.1.31 Assisted reproductive technologies

A meta-analysis with 106,338 participants and 4,990 cases depicted that assisted reproductive technologies can reduce the risk of postpartum depression (OR: 0.83, 95% CI: 0.71, 0.97) (53) (Figure 2).

##### 3.2.1.32 Postpartum hemorrhage

Tung's study with 4,990 cases and 46,508 participants showed that postpartum hemorrhage can increase the risk of postpartum depression (RR: 1.28, 95% CI: 1.13, 1.44) (54) (Figure 2).

##### 3.2.1.33 Polycystic ovary syndrome

Schoretsanitis's study with six cohorts studies showed that polycystic ovary syndrome increases the risk of postpartum depression (OR: 1.45, 95% CI: 1.18, 1.79) (55) (Figure 2).

##### 3.2.1.34 Maternal alcohol

Qiu's study with six cohorts studies showed that maternal alcohol can increase the risk of postpartum depression (OR: 1.21, 95% CI: 1.04, 1.41) (56) (Figure 2).

##### 3.2.1.35 Mother's personality

A meta-analysis with 50,377 participants depicted that neuroticism women are more prone to postpartum depression (OR: 1.37, 95% CI: 1.22, 1.53) (57) (Figure 2).

##### 3.2.1.36 Substance abuse

A meta-analysis with 485,305 participants and 16,778 cases depicted that substance abuse can increase the risk of postpartum depression (OR: 3.67, 95% CI: 2.31, 5.85) (58) (Figure 2).

##### 3.2.1.37 Postpartum pain

Lu's study with eight cohorts studies showed that postpartum pain can increase the risk of postpartum depression (OR: 1.29, 95% CI: 1.10, 1.52) (59) (Figure 2).

##### 3.2.1.38 Perinatal pain

Mo's study with 10 cohorts studies showed that perinatal pain can increase the risk of postpartum depression (OR: 1.43, 95% CI: 1.23, 1.67) (60) (Figure 2).

##### 3.2.1.39 Thyroid autoimmunity

A meta-analysis with 2,932 participants and 449 cases depicted that TPOAb- increases the risk of postpartum depression (OR: 1.49, 95% CI: 1.11, 2.00) (61) (Figure 2).

##### 3.2.1.40 Adverse childhood experiences

Mo's study with 21,011 participants showed that adverse childhood experiences can increase the risk of postpartum depression (OR: 2.31, 95% CI: 2.04, 2.63) (62) (Figure 2).

#### 3.2.2 Non-significant risk factors

This study found that PM2.5, road traffic noise (≥65 dB) (26), infertility treatment (0–3 and 3–6 months after delivery) (41), prenatal exercise (63), epidural analgesia (64), neuraxial analgesia during labor (65), air pollution (NO2, PM2.5, PM10) (26), public space (NDVI, green access, blue access) (26) has no significant impact on the risk of postpartum depression (Table 2).

### 3.3 Heterogeneity

The reanalysis revealed that 68% of the assessed risk factors exhibited significant heterogeneity, with *I*^2^ values exceeding 50% or Cochran's *Q*-test *P*-values below 0.1. This variability may arise from factors such as study setting, region, ethnicity, gender, age, study quality, design, sample size, follow-up duration, and adjustments for confounders. Furthermore, 1.9% of studies did not report heterogeneity assessment results (Table 2).

### 3.4 Assessment of risk of bias

Egger's test evaluated publication bias for 47.2% of the identified risk factors, identifying bias in seven factors: Breastfeeding support, 25(OH)D level, maternal birth complications, epidural analgesia, neuroticism, postpartum pain, adverse childhood experiences. No significant publication bias was detected for other outcomes, or bias assessments were not reported (Table 2).

### 3.5 AMSTAR score

The identified risk factors had a median AMSTAR score of 7.83 (range: 6–10). Comprehensive AMSTAR scores for each outcome are presented in Supplementary Table S2.

### 3.6 Grade rating

Only parenting interventions was assigned a high grade, doula delivery, skin-to-skin contact, postpartum physical activity were assigned a moderate grade, as they were RCTs. Other factors were assigned low or very low grades, primarily due to their cohort or case-control design (Supplementary Table S3). The evidence class classifications and corresponding GRADE certainty ratings for all risk factors are summarized in Table 2.

## 4 Discussion

This umbrella review synthesized evidence from 77 meta-analyses, identifying 53 risk factors for postpartum depression (PPD), of which 40 were statistically significant. Among the risk factors with the strongest associations and highest evidence levels were unintended pregnancy, intimate partner violence, poor social support, a history of depression, and sleep disorders. Protective factors included postpartum physical activity, parenting interventions, doula support, and breastfeeding support. Using AMSTAR and GRADE tools, we determined that most evidence was of low or very low certainty, with only a few randomized controlled trials providing moderate to high certainty. RCTs are more likely to receive higher GRADE ratings because randomization minimizes confounding, reduces selection bias, and provides stronger causal inference than observational designs such as cohort or case–control studies. These methodological strengths improve internal validity and explain why intervention-related factors supported by RCTs, such as parenting interventions and doula support, achieved higher certainty levels. Notably, unintended pregnancy was identified as a Class I risk factor, with a robust association with PPD (OR: 1.55), consistent across multiple meta-analyses. Moreover, social determinants, such as immigration status and marital dissatisfaction, emerged as influential contributors to PPD vulnerability, highlighting the complex interplay of psychosocial and biological risk factors.

Our findings reinforce previous umbrella reviews identifying psychosocial stressors as core drivers of PPD. Hutchens and Kearney (66) emphasized that lack of social support, history of abuse, and prenatal depression were among the most consistently replicated risk factors. In line with this, our analysis confirmed strong associations between intimate partner violence (OR: 2.50), poor social support (RR: 3.57), and prior depression (OR: 3.09) with elevated PPD risk. These outcomes align with Gastaldon et al. (67), who classified violent experiences and premenstrual syndrome as highly suggestive risk factors. Furthermore, our review corroborated evidence showing that women exposed to stressful life events during pregnancy (OR: 1.82) and those with a family history of psychiatric disorders (OR: 2.08) were at greater risk of developing PPD. Such findings emphasize the need for early psychosocial assessments during antenatal care to identify at-risk individuals (68, 69).

Biological and obstetric factors also emerged as important contributors, although with more modest effect sizes and generally lower evidence quality. Our analysis found that gestational diabetes (OR: 1.59), anemia (OR: 1.53), cesarean section (RR: 1.12), and vitamin D deficiency (OR: 3.67) significantly increased the risk of PPD. These findings are partially supported by previous literature. Aghajafari et al. (6) reported a consistent link between low 25(OH)D levels and PPD, while Gastaldon et al. (67) rated the evidence for gestational diabetes and anemia as “suggestive” and “weak,” respectively. Moreover, maternal complications during childbirth, such as postpartum hemorrhage and delivery by cesarean section, can lead to traumatic birth experiences, which may heighten the risk of depressive symptoms in the postpartum period (4). Interestingly, genetic factors such as five-HTTLPR polymorphism also showed a modest protective effect (OR 0.54), indicating the relevance of gene-environment interactions in PPD etiology (70).

Although our review draws upon evidence from diverse global populations, sociocultural, economic, and healthcare system differences may shape both the magnitude and interpretation of these risk factors. For example, the influence of social support differs across regions due to variations in family structure, community networks, and expectations regarding postpartum care. Similarly, immigration status may involve distinct stressors across countries, including legal vulnerability, language barriers, and unequal access to perinatal mental health services. Differences in healthcare infrastructure, screening practices, and stigma surrounding mental health may also influence the expression and identification of PPD-related risk factors. Recognizing this contextual variability is crucial for interpreting the generalizability of the findings and tailoring prevention strategies to specific sociocultural contexts.

Several lifestyle and intervention-related factors showed promise in mitigating PPD risk. Postpartum physical activity (SMD: −0.42), doula-assisted deliveries (RR: 0.36), breastfeeding support (MD: −2.11), and parenting interventions (SMD: −0.34) were associated with decreased severity or incidence of PPD. These findings are consistent with Dipietro et al. (71), who concluded that physical activity during the perinatal period offers significant psychological benefits, including reduced depressive symptoms. Additionally, our findings echo the work of Adjie et al. (5), who found that breastfeeding support not only enhances infant health but also reduces maternal emotional distress. Importantly, these modifiable behavioral interventions offer non-pharmacological pathways for prevention and treatment and should be prioritized in maternal health strategies. However, evidence quality varied, with only a few outcomes supported by high-quality RCTs. For instance, the GRADE assessment assigned high confidence only to parenting interventions and moderate confidence to doula support and physical activity interventions, emphasizing the need for more rigorous trials.

Despite these comprehensive findings, this review has several limitations. First, the majority of included studies were observational, which inherently limits causal inference due to potential confounding and bias. Many outcomes showed significant heterogeneity (*I*^2^ > 50%), reflecting variability in study populations, measurement tools, and study designs. Moreover, the certainty of evidence, as assessed by GRADE, was predominantly low or very low, with only a few high-quality randomized controlled trials. Egger's test revealed publication bias in several outcomes, including breastfeeding support and maternal birth complications, potentially inflating effect estimates. Another limitation was the exclusion of non-English literature, which may bias the findings toward English-speaking populations. Lastly, some included meta-analyses were outdated or overlapped in primary studies, despite our effort to select the most comprehensive and recent ones. To critically interpret these findings, it is necessary to examine how the identified limitations affect the certainty of the conclusions. For example, the strong association observed for unintended pregnancy (OR: 1.55) contrasts with its low GRADE rating, largely because reliance on large observational cohorts yields stable estimates yet remains susceptible to residual confounding, misclassification of pregnancy intention, and socioeconomic gradients that are difficult to fully adjust for. Thus, the apparent strength of the association should not be interpreted as high certainty. Similarly, many risk factors with low or very low GRADE ratings-including gestational diabetes, anemia, cesarean section, and vitamin D deficiency-continued to demonstrate consistent effect directions across diverse populations. Although these findings cannot establish causality, they may serve as indicators for enhanced clinical surveillance rather than definitive causal determinants. For the non-significant risk factors identified in our review (e.g., air pollution, prenatal exercise, epidural analgesia), previous studies have also reported inconsistent or null associations, suggesting that these exposures may exert weaker effects, be context-dependent, or suffer from measurement variability. These prior findings support the plausibility of non-significant associations in our results and highlight the need for standardized exposure assessment and adequately powered cohort studies. This interpretation aligns with recent high-quality umbrella reviews, such as Gastaldon et al. (2022) (67) and Kim et al. (2022) (72), both of which reported that most PPD-related associations are credible yet lack high-certainty evidence. Our results therefore fit within the broader evidence landscape and extend prior evidence by incorporating more recent meta-analyses and a wider range of psychosocial and obstetric factors. Moreover, the high heterogeneity observed in many pooled estimates suggests that the magnitude of risk likely varies across cultural contexts, assessment tools, and healthcare systems, highlighting the need for standardized diagnostic criteria and harmonized measurement approaches without undermining the associations themselves. Taken together, these considerations underscore that low-certainty evidence does not diminish the practical value of many identified risk factors and emphasizes the need to interpret these associations as indicators of vulnerability that may inform clinical decision-making rather than as definitive causal relationships. In addition, given the consistent associations reported for intimate partner violence and inadequate social support, our findings indicate several actionable implications for screening and public health practice. Routine antenatal and postnatal screening for interpersonal violence and deficits in social support could be incorporated into primary maternal healthcare, supported by structured referral pathways to psychological counseling, social work services, or community-based support programs. Public health policies that strengthen social support systems-such as community mother–baby programs, partner-engaged interventions, and culturally tailored services for immigrant women-may help reduce PPD risk. These measures provide feasible strategies for early identification and prevention, especially in resource-limited settings. Future research should focus on large-scale prospective studies and high-quality RCTs to confirm these associations and explore underlying mechanisms.

## 5 Conclusion

This umbrella review presents a comprehensive synthesis of current evidence on risk factors for postpartum depression, integrating data from 77 meta-analyses and over 50 individual risk factors. We identified several high-impact and clinically relevant contributors to PPD, including unintended pregnancy, intimate partner violence, poor social support, and a personal or family history of depression. These findings reaffirm the complex etiology of PPD, underscoring the role of both psychosocial and biological determinants.

Importantly, several modifiable factors such as postpartum physical activity, breastfeeding support, doula care, and parenting interventions were associated with reduced PPD risk or symptom severity, highlighting key opportunities for non-pharmacological prevention and intervention. These interventions, particularly those supported by randomized trials, should be prioritized in clinical guidelines and maternal mental health programs. Additionally, the influence of structural determinants such as immigration status, socioeconomic adversity, and marital satisfaction should not be overlooked when designing public health responses.

Nevertheless, this review revealed that the majority of evidence is derived from observational studies with substantial heterogeneity and low methodological rigor. Future studies must address these gaps by incorporating standardized diagnostic criteria, diverse populations, and robust analytical designs. Ultimately, enhancing the quality of evidence will be essential for refining screening tools, tailoring interventions, and informing policies aimed at improving postpartum mental health outcomes globally.

## Data Availability

The original contributions presented in the study are included in the article/Supplementary material, further inquiries can be directed to the corresponding author.
