# Supplementary material for: Risk factors for postpartum depression: an umbrella review
Source: Front Public Health. 2026 Jan 22;13:1714668. doi: 10.3389/fpubh.2025.1714668 (PMC12872911; doi:10.3389/fpubh.2025.1714668)
Supplement: Supplementary file 1 [file Table_1.docx]

| **Table S1** The searching strategy | | |
| --- | --- | --- |
| Database | Search strategy | Number |
| Cochrane | #1 (Postpartum Period):ti,ab,kw OR (Period, Postpartum):ti,ab,kw OR (Postpartum):ti,ab,kw OR (Postpartum Women):ti,ab,kw OR (Women, Postpartum):ti,ab,kw  #2 (Puerperium):ti,ab,kw  #3 #1 OR #2  #4 (Depression):ti,ab,kw OR (Depressive Symptoms):ti,ab,kw OR (Depressive Symptom):ti,ab,kw OR (Symptom, Depressive):ti,ab,kw OR (Emotional Depression):ti,ab,kw  #5 (Depression, Emotional):ti,ab,kw  #6 #4 OR #5  #7 (meta-analysis):ti,ab,kw OR (systematic review):ti,ab,kw  #8 #3 AND #6 AND #7 | 83 |
| Embase | #20 #8 AND #16 AND #19  #19 #17 OR #18  #18 'systematic review':ab,kw,ti  #17 'meta analysis':ab,kw,ti  #16 #9 OR #10 OR #11 OR #12 OR #13 OR #14 OR #15  #15 'depression, emotional':ab,kw,ti  #14 'emotional depression':ab,kw,ti  #13 'symptom, depressive':ab,kw,ti  #12 'depressive symptom':ab,kw,ti  #11 'depressive symptoms':ab,kw,ti  #10 depression:ab,kw,ti  #9 'depression'/exp  #8 #1 OR #2 OR #3 OR #4 OR #5 OR #6 OR #7  #7 'puerperium':ab,kw,ti  #6 'women, postpartum':ab,kw,ti  #5 'postpartum women':ab,kw,ti  #4 'postpartum':ab,kw,ti  #3 'period, postpartum':ab,kw,ti  #2 'postpartum period':ab,kw,ti  #1 'postpartum period'/exp | 1005 |
| PubMed | (((((((Postpartum Period) OR (Period, Postpartum)) OR (Postpartum)) OR (Postpartum Women)) OR (Women, Postpartum)) OR (Puerperium)) AND ((((((Depression) OR (Depressive Symptoms)) OR (Depressive Symptom)) OR (Symptom, Depressive)) OR (Emotional Depression)) OR (Depression, Emotional))) AND ((meta-analysis) OR (systematic review)) | 1035 |
| Web of science | 1 "(((((ALL=(Postpartum Period)) OR ALL=(Period, Postpartum)) OR ALL=(Postpartum)) OR ALL=(Postpartum Women)) OR ALL=(Women, Postpartum)) OR ALL=(Puerperium) "  2 "(((((ALL=(Depression)) OR ALL=(Depressive Symptoms)) OR ALL=(Depressive Symptom)) OR ALL=(Symptom, Depressive)) OR ALL=(Emotional Depression)) OR ALL=(Depression, Emotional) "  3 "(ALL=(meta-analysis)) OR ALL=(systematic review) "  4 "#3 AND #2 AND #1 " | 1488 |

| **Table S2** AMSTAR score of risk factors associated with postpartum depression | | | | | | | | | | | | | | | | | | | | | | | | | | | | | | | | | |
| --- | --- | --- | --- | --- | --- | --- | --- | --- | --- | --- | --- | --- | --- | --- | --- | --- | --- | --- | --- | --- | --- | --- | --- | --- | --- | --- | --- | --- | --- | --- | --- | --- | --- |
| **Outcome** | | **Category** | | **Assessed with** | | | **Author** | **Year** | | **A priori design provided** | | | **Duplicate study selection & data extraction** | | **At least two electronic databases searched** | **Status of publication used as an inclusion criterion** | | **List of included and excluded studies provided** | | **Characteristics of included studies provided** | | | **Scientific quality of included studies assessed** | **Scientific quality of the included studies used appropriately to form conclusions** | | | **Appropriate methods to combine studies** | **Publication bias assessed** | | **Conflict of interest included** | | **Total AMSTAR Score** | |
| Unintended pregnancy | | Postpartum depression risk | | Unintended vs intended pregnancy | | | Nelson | 2022 | | 1 | | | 1 | | 1 | 0 | | 1 | | 1 | | | 0 | 1 | | | 1 | 1 | | 1 | | 9 | |
| Parenting Interventions | | score | | with vs without | | | Adina | 2022 | | 1 | | | 1 | | 1 | 0 | | 1 | | 1 | | | 1 | 0 | | | 1 | 1 | | 1 | | 9 | |
| Breastfeeding support | | EPDS score | | BF intervention vs Standard care | | | Adjie | 2024 | | 1 | | | 1 | | 1 | 0 | | 1 | | 1 | | | 1 | 0 | | | 1 | 1 | | 1 | | 9 | |
| COVID-19 | | Postpartum depression risk | | with vs without | | | Caffieri | 2023 | | 1 | | | 1 | | 1 | 0 | | 1 | | 1 | | | 1 | 1 | | | 1 | 1 | | 1 | | 10 | |
| 25(OH)D level | | Postpartum depression risk | | <50 nmol/l vs ≥50 nmol/l | | | Wang | 2018 | | 0 | | | 1 | | 1 | 0 | | 1 | | 1 | | | 1 | 0 | | | 1 | 1 | | 1 | | 8 | |
| gestational diabetes | | Postpartum depression risk | | NA | | | Azami | 2019 | | 0 | | | 1 | | 1 | 0 | | 1 | | 1 | | | 1 | 0 | | | 1 | 1 | | 1 | | 8 | |
| immigrant women | | Postpartum depression risk | | immigrant women vs native women | | | Falah-Hassani | 2015 | | 0 | | | 1 | | 1 | 0 | | 1 | | 1 | | | 0 | 0 | | | 1 | 1 | | 1 | | 7 | |
| Intimate Partner violence | | Postpartum depression risk | | NA | | | Wei | 2024 | | 0 | | | 1 | | 1 | 0 | | 1 | | 1 | | | 0 | 0 | | | 1 | 1 | | 1 | | 7 | |
| 5 HTTLPR polymorphism | | Postpartum depression risk | | patients vs healthy | | | Li | 2020 | | 0 | | | 1 | | 1 | 0 | | 1 | | 1 | | | 1 | 0 | | | 1 | 1 | | 1 | | 8 | |
| anemia | | Postpartum depression risk | | NA | | | Kang | 2020 | | 0 | | | 1 | | 1 | 0 | | 1 | | 1 | | | 1 | 0 | | | 1 | 1 | | 1 | | 8 | |
| caesarean section | | Postpartum depression risk | | caesarean section vs natural vaginal delivery | | | Ning | 2024 | | 1 | | | 1 | | 1 | 0 | | 1 | | 1 | | | 1 | 0 | | | 1 | 1 | | 1 | | 9 | |
| poor social support | | Postpartum depression risk | | poor vs strong social support | | | Zeleke | 2021 | | 1 | | | 1 | | 1 | 0 | | 1 | | 1 | | | 1 | 0 | | | 1 | 1 | | 1 | | 9 | |
| sleep disorders | | Postpartum depression risk | | sleep disorders vs control | | | Li | 2023 | | 1 | | | 1 | | 1 | 0 | | 1 | | 1 | | | 1 | 0 | | | 1 | 1 | | 1 | | 9 | |
| PM2.5 | | Postpartum depression risk | | Single exposure | | | Cadman | 2024 | | 0 | | | 0 | | 1 | 0 | | 1 | | 1 | | | 0 | 0 | | | 1 | 0 | | 1 | | 5 | |
| PM10 | | Postpartum depression risk | | 10μg/m3 change | | | Pourhoseini | 2024 | | 0 | | | 1 | | 1 | 0 | | 1 | | 1 | | | 1 | 0 | | | 1 | 0 | | 1 | | 7 | |
| Road traffic noise | | Postpartum depression risk | | Lden>=65 dB | | | Cadman | 2024 | | 0 | | | 0 | | 1 | 0 | | 1 | | 1 | | | 0 | 0 | | | 1 | 0 | | 1 | | 5 | |
| History of depression | | Postpartum depression risk | | with vs without | | | Liu | 2022 | | 1 | | | 1 | | 1 | 0 | | 1 | | 1 | | | 1 | 0 | | | 1 | 1 | | 1 | | 9 | |
| maternal birth complacations | | Postpartum depression risk | | with vs without | | | Cárdenas | 2025 | | 0 | | | 1 | | 1 | 0 | | 1 | | 1 | | | 0 | 1 | | | 1 | 1 | | 1 | | 7 | |
| doula delivery | | score | | doula delivery vs control | | | Gong | 2017 | | 0 | | | 1 | | 1 | 0 | | 1 | | 1 | | | 1 | 0 | | | 1 | 0 | | 0 | | 6 | |
| Prenatal smoking | | Postpartum depression risk | | with vs without | | | Chen | 2019 | | 0 | | | 1 | | 1 | 0 | | 1 | | 1 | | | 1 | 0 | | | 1 | 1 | | 1 | | 8 | |
| infertility treatment | | Postpartum depression risk | | with vs without | | | Chen | 2019 | | 0 | | | 1 | | 1 | 0 | | 1 | | 1 | | | 1 | 0 | | | 1 | 0 | | 1 | | 7 | |
| Skin-to-skin contact | | Postpartum depression risk | | with vs without | | | Cong | 2021 | | 0 | | | 1 | | 1 | 0 | | 1 | | 1 | | | 1 | 0 | | | 1 | 1 | | 1 | | 8 | |
| obese women | | Postpartum depression risk | | obese women vs normal weight control | | | Molyneaux | 2014 | | 1 | | | 1 | | 1 | 0 | | 1 | | 1 | | | 0 | 0 | | | 1 | 1 | | 0 | | 7 | |
| prenatal exercise | | score | | with vs without | | | Davenport | 2018 | | 1 | | | 1 | | 1 | 0 | | 1 | | 0 | | | 0 | 1 | | | 0 | 1 | | 1 | | 7 | |
| postpartum physical activity | | score | | with vs without | | | Deprato | 2024 | | 1 | | | 1 | | 1 | 0 | | 1 | | 1 | | | 1 | 1 | | | 1 | 1 | | 1 | | 10 | |
| Prenatal stressful life events | | Postpartum depression risk | | with vs without | | | Ding | 2023 | | 1 | | | 1 | | 1 | 0 | | 1 | | 1 | | | 1 | 0 | | | 1 | 1 | | 1 | | 9 | |
| Preterm birth | | Postpartum depression risk | | preterm infants vs full-term | | | Eduardo | 2019 | | 0 | | | 1 | | 1 | 0 | | 1 | | 1 | | | 1 | 0 | | | 1 | 0 | | 1 | | 7 | |
| Urinary | | Postpartum depression risk | | with vs without | | | Gallego-Gómez | 2024 | | 1 | | | 1 | | 1 | 0 | | 1 | | 1 | | | 1 | 0 | | | 1 | 0 | | 1 | | 8 | |
| epidural analgesia | | Postpartum depression risk | | with vs without | | | Ghanbari-Homaie | 2024 | | 0 | | | 1 | | 1 | 0 | | 1 | | 1 | | | 0 | 0 | | | 1 | 1 | | 1 | | 7 | |
| Family History of Psychiatric Disorders | | Postpartum depression risk | | with vs without | | | Zacher Kjeldse | 2022 | | 1 | | | 1 | | 1 | 0 | | 1 | | 1 | | | 1 | 1 | | | 1 | 1 | | 1 | | 10 | |
| female | | Postpartum depression risk | | female vs male infant | | | Ye | 2020 | | 0 | | | 1 | | 1 | 0 | | 1 | | 1 | | | 1 | 0 | | | 1 | 1 | | 1 | | 8 | |
| Violence | | Postpartum depression risk | | with vs without | | | Zhang | 2019 | | 0 | | | 1 | | 1 | 0 | | 1 | | 1 | | | 0 | 0 | | | 1 | 1 | | 1 | | 7 | |
| marital satisfaction | | Postpartum depression risk | | Low vs high | | | Wei | 2025 | | 1 | | | 1 | | 1 | 0 | | 1 | | 1 | | | 1 | 0 | | | 1 | 1 | | 1 | | 9 | |
| single/divorced/separated/widowed/unmarried | | Postpartum depression risk | | - | | | Wei | 2025 | | 1 | | | 1 | | 1 | 0 | | 1 | | 1 | | | 1 | 0 | | | 1 | 1 | | 1 | | 9 | |
| winter | | Postpartum depression risk | | winter vs other seasons | | | Tung | 2022 | | 0 | | | 1 | | 1 | 0 | | 1 | | 1 | | | 1 | 0 | | | 1 | 1 | | 1 | | 8 | |
| Assisted reproductive technologies | | Postpartum depression risk | | with vs without | | | Zhao | 2024 | | 0 | | | 1 | | 1 | 0 | | 1 | | 1 | | | 0 | 0 | | | 1 | 0 | | 1 | | 6 | |
| Postpartum hemorrhage | | Postpartum depression risk | | with vs without | | | Schoretsanitis | 2024 | | 1 | | | 1 | | 1 | 0 | | 1 | | 1 | | | 1 | 0 | | | 1 | 1 | | 1 | | 9 | |
| Polycystic ovary syndrome | | Postpartum depression risk | | with vs without | | | Schoretsanitis | 2022 | | 1 | | | 1 | | 1 | 0 | | 1 | | 1 | | | 0 | 1 | | | 1 | 1 | | 1 | | 9 | |
| maternal alcohol | | Postpartum depression risk | | with vs without | | | Qiu | 2022 | | 0 | | | 1 | | 1 | 0 | | 1 | | 1 | | | 1 | 0 | | | 1 | 1 | | 1 | | 8 | |
| neuroticism | | Postpartum depression risk | | with vs without | | | Neda-Stepan | 2024 | | 1 | | | 1 | | 1 | 0 | | 1 | | 1 | | | 1 | 0 | | | 1 | 1 | | 1 | | 9 | |
| substance abuse | | Postpartum depression risk | | with vs without | | | Pacho | 2023 | | 1 | | | 1 | | 1 | 0 | | 1 | | 1 | | | 1 | 0 | | | 1 | 1 | | 1 | | 9 | |
| Postpartum pain | | Postpartum depression risk | | with vs without | | | Lu | 2024 | | 0 | | | 1 | | 1 | 0 | | 1 | | 1 | | | 1 | 1 | | | 1 | 1 | | 1 | | 9 | |
| hyroid autoimmunity | | Postpartum depression risk | | TPOAb- vs TPOAb+ | | | Minaldi | 2020 | | 1 | | | 1 | | 1 | 0 | | 1 | | 1 | | | 1 | 0 | | | 1 | 1 | | 1 | | 9 | |
| perinatal pain | | Postpartum depression risk | | with vs without | | | Mo | 2022 | | 0 | | | 1 | | 1 | 0 | | 1 | | 1 | | | 1 | 0 | | | 1 | 1 | | 1 | | 8 | |
| Neuraxial analgesia during labor | | Postpartum depression risk | | with vs without | | | Li | 2023 | | 1 | | | 1 | | 1 | 0 | | 1 | | 1 | | | 1 | 0 | | | 1 | 1 | | 0 | | 8 | |
| Adverse childhood experiences | | Postpartum depression risk | | with vs without | | | Fu | 2024 | | 1 | | | 1 | | 1 | 0 | | 1 | | 1 | | | 1 | 0 | | | 1 | 1 | | 1 | | 9 | |
| NO2 | | Postpartum depression risk | | with vs without | | | Cadman | 2024 | | 0 | | | 1 | | 1 | 0 | | 1 | | 1 | | | 0 | 0 | | | 1 | 0 | | 1 | | 6 | |
| PM2.5 | | Postpartum depression risk | | with vs without | | | Cadman | 2024 | | 0 | | | 1 | | 1 | 0 | | 1 | | 1 | | | 0 | 0 | | | 1 | 0 | | 1 | | 6 | |
| PM10 | | Postpartum depression risk | | with vs without | | | Cadman | 2024 | | 0 | | | 1 | | 1 | 0 | | 1 | | 1 | | | 0 | 0 | | | 1 | 0 | | 1 | | 6 | |
| NDVI | | Postpartum depression risk | | with vs without | | | Cadman | 2024 | | 0 | | | 1 | | 1 | 0 | | 1 | | 1 | | | 0 | 0 | | | 1 | 0 | | 1 | | 6 | |
| Green access | | Postpartum depression risk | | with vs without | | | Cadman | 2024 | | 0 | | | 1 | | 1 | 0 | | 1 | | 1 | | | 0 | 0 | | | 1 | 0 | | 1 | | 6 | |
| Blue access | | Postpartum depression risk | | with vs without | | | Cadman | 2024 | | 0 | | | 1 | | 1 | 0 | | 1 | | 1 | | | 0 | 0 | | | 1 | 0 | | 1 | | 6 | |
| Table S3 GRADE rating of risk factors associated with postpartum depression | | | | | | | | | | | | | | | | | | | | | | | | | | | | | | | | | |
| **Study** | **Intervention** | | **Control** | | **Diseases** | **Outcomes** | | | **No. of studies** | | **RCT** | **Cohort** | | **case-control/cross-sectional** | | | **Risk of Bias** | | **Inconsistency（<50%,no serious)** | | **Indirectness** | **Imprecision** | | | **Publication bias** | **Plausible confounding** | | | **Magnitude of effect** | | **Dose-response gradient** | | **Quality** |
| Nelson 2022 | Unintended Pregnancy | | Unintended vs intended pregnancy | | Postpartum depression | Postpartum depression risk | | | 10 | | 0 | 4 | | 6 | | | serious | | no serious | | no serious | no serious | | | no serious | would not reduce effect | | | no | | no | | very low |
| Adina 2022 | Parenting Interventions | | with vs without | | Postpartum depression | score | | | 15 | | 15 | 0 | | 0 | | | no serious | | no serious | | no serious | no serious | | | no serious | would not reduce effect | | | no | | no | | high |
| Adjie 2024 | Breastfeeding support | | BF intervention vs Standard care | | Postpartum depression | EPDS score | | | 10 | | 10 | 0 | | 0 | | | serious | | serious | | no serious | no serious | | | serious | would not reduce effect | | | no | | no | | very low |
| Caffieri 2023 | COVID-19 | | with vs without | | Postpartum depression | Postpartum depression risk | | | 54 | | 0 | 0 | | 54 | | | no serious | | serious | | no serious | no serious | | | no serious | would not reduce effect | | | no | | no | | low |
| Wang 2018 | 25(OH)D level | | <50 nmol/l vs ≥50 nmol/l | | Postpartum depression | Postpartum depression risk | | | 4 | | 0 | 4 | | 0 | | | no serious | | serious | | no serious | no serious | | | serious | would not reduce effect | | | no | | no | | very low |
| Azami 2019 | gestational diabetes | | NA | | Postpartum depression | Postpartum depression risk | | | 18 | | 0 | 5 | | 3 | | | no serious | | serious | | no serious | no serious | | | no serious | would not reduce effect | | | no | | no | | very low |
| Falah-Hassani 2015 | immigrant women | | immigrant women vs native women | | Postpartum depression | Postpartum depression risk | | | 5 | | 0 | 2 | | 3 | | | serious | | serious | | no serious | no serious | | | no serious | would not reduce effect | | | no | | no | | very low |
| Wei 2024 | Intimate Partner violence | | NA | | Postpartum depression | Postpartum depression risk | | | 76 | | 0 | 34 | | 42 | | | serious | | serious | | no serious | no serious | | | no serious | would not reduce effect | | | yes | | no | | very low |
| Li 2020 | 5 HTTLPR polymorphism | | patients vs healthy | | Postpartum depression | Postpartum depression risk | | | 6 | | 0 | 0 | | 6 | | | no serious | | serious | | no serious | no serious | | | no serious | would not reduce effect | | | no | | no | | very low |
| Kang 2020 | anemia | | NA | | Postpartum depression | Postpartum depression risk | | | 7 | | 0 | NA | | NA | | | no serious | | serious | | no serious | no serious | | | no serious | would not reduce effect | | | no | | no | | very low |
| Ning 2024 | caesarean section | | caesarean section vs natural vaginal delivery | | Postpartum depression | Postpartum depression risk | | | 8 | | 0 | 6 | | 2 | | | no serious | | no serious | | no serious | no serious | | | no serious | would not reduce effect | | | no | | no | | low |
| Zeleke 2021 | poor social support | | poor vs strong social support | | Postpartum depression | Postpartum depression risk | | | 3 | | 0 | 0 | | 3 | | | no serious | | serious | | no serious | no serious | | | no serious | would not reduce effect | | | yes | | no | | low |
| Li 2023a | sleep disorders | | sleep disorders vs control | | Postpartum depression | Postpartum depression risk | | | 16 | | 0 | 16 | | 0 | | | no serious | | serious | | no serious | no serious | | | serious | would not reduce effect | | | no | | no | | very low |
| Cadman 2024 | PM2.5 | | Single exposure | | Postpartum depression | Postpartum depression risk | | | 12 | | 0 | 12 | | 0 | | | serious | | no serious | | no serious | no serious | | | serious | would not reduce effect | | | no | | no | | very low |
| Pourhoseini 2024 | PM10 | | 10μg/m3 change | | Postpartum depression | Postpartum depression risk | | | 2 | | 0 | 2 | | 0 | | | no serious | | serious | | no serious | no serious | | | serious | would not reduce effect | | | no | | no | | very low |
| Cadman 2024 | Road traffic noise | | Lden>=65 dB | | Postpartum depression | Postpartum depression risk | | | 11 | | 0 | 11 | | 0 | | | serious | | no serious | | no serious | no serious | | | serious | would not reduce effect | | | no | | no | | very low |
| Liu 2022 | History of depression | | with vs without | | Postpartum depression | Postpartum depression risk | | | 4 | | 0 | 2 | | 2 | | | no serious | | serious | | no serious | no serious | | | no serious | would not reduce effect | | | yes | | no | | low |
| Cárdenas 2025 | maternal birth complacations | | with vs without | | Postpartum depression | Postpartum depression risk | | | 61 | | 0 | NA | | NA | | | serious | | serious | | no serious | no serious | | | serious | would not reduce effect | | | no | | no | | very low |
| Gong 2017 | doula delivery | | doula delivery vs control | | Postpartum depression | Postpartum depression risk | | | 13 | | 13 | 0 | | 0 | | | no serious | | no serious | | no serious | no serious | | | serious | would not reduce effect | | | no | | no | | moderate |
| Chen 2019a | Prenatal smoking | | with vs without | | Postpartum depression | Postpartum depression risk | | | 13 | | 0 | 4 | | 4 | | | no serious | | serious | | no serious | no serious | | | no serious | would not reduce effect | | | no | | no | | very low |
| Chen 2019b | infertility treatment | | with vs without | | Postpartum depression | Postpartum depression risk | | | 11/4/2 | | 0 | NA | | NA | | | no serious | | no serious | | no serious | no serious | | | serious | would not reduce effect | | | no | | no | | low |
| Cong 2021 | Skin-to-skin contact | | with vs without | | Postpartum depression | score | | | 6 | | 6 | 0 | | 0 | | | no serious | | serious | | no serious | no serious | | | serious | would not reduce effect | | | no | | no | | moderate |
| Molyneaux 2014 | obese women | | obese women vs normal weight control | | Postpartum depression | Postpartum depression risk | | | 15 | | 0 | NA | | NA | | | no serious | | no serious | | no serious | no serious | | | serious | would not reduce effect | | | no | | no | | low |
| Davenport 2018 | prenatal exercise | | with vs without | | Postpartum depression | score | | | 4 | | NA | NA | | NA | | | serious | | no serious | | no serious | no serious | | | serious | would not reduce effect | | | no | | no | | very low |
| Deprato 2024 | postpartum physical activity | | with vs without | | Postpartum depression | score | | | 35 | | 35 | 0 | | 0 | | | no serious | | serious | | no serious | no serious | | | serious | would not reduce effect | | | no | | no | | moderate |
| Ding 2023 | Prenatal stressful life events | | with vs without | | Postpartum depression | Postpartum depression risk | | | 17 | | 0 | 17 | | 0 | | | no serious | | serious | | no serious | no serious | | | no serious | would not reduce effect | | | no | | no | | very low |
| Eduardo 2019 | Preterm birth | | preterm infants vs full-term | | Postpartum depression | Postpartum depression risk | | | 12 | | 0 | 4 | | 8 | | | no serious | | serious | | no serious | no serious | | | serious | would not reduce effect | | | no | | no | | very low |
| Gallego-Gómez 2024 | Urinary | | with vs without | | Postpartum depression | Postpartum depression risk | | | 11 | | 0 | 8 | | 4 | | | no serious | | serious | | no serious | no serious | | | serious | would not reduce effect | | | no | | no | | very low |
| Ghanbari-Homaie 2024 | epidural analgesia | | with vs without | | Postpartum depression | Postpartum depression risk | | | 3 | | 0 | 3 | | 0 | | | serious | | serious | | no serious | no serious | | | serious | would not reduce effect | | | no | | no | | very low |
| Zacher Kjeldse 2022 | Family History of Psychiatric Disorders | | with vs without | | Postpartum depression | Postpartum depression risk | | | 25 | | 0 | 23 | | 2 | | | no serious | | serious | | no serious | no serious | | | serious | would not reduce effect | | | no | | no | | very low |
| Ye 2020 | female | | female vs male infant | | Postpartum depression | Postpartum depression risk | | | 25 | | 0 | 19 | | 6 | | | no serious | | serious | | no serious | no serious | | | no serious | would not reduce effect | | | no | | no | | very low |
| Zhang 2019 | Violence | | with vs without | | Postpartum depression | Postpartum depression risk | | | 32 | | 0 | 26 | | 6 | | | serious | | serious | | no serious | no serious | | | no serious | would not reduce effect | | | no | | no | | very low |
| Wei 2025 | marital satisfaction | | Low vs high | | Postpartum depression | Postpartum depression risk | | | 5 | | 0 | 5 | | 0 | | | no serious | | serious | | no serious | no serious | | | no serious | would not reduce effect | | | yes | | no | | low |
| Wei 2025 | single/divorced/separated/widowed/unmarried | | - | | Postpartum depression | Postpartum depression risk | | | 11 | | 0 | 11 | | 0 | | | no serious | | serious | | no serious | no serious | | | no serious | would not reduce effect | | | no | | no | | very low |
| Tung 2022 | winter | | winter vs other seasons | | Postpartum depression | Postpartum depression risk | | | 5 | | 0 | 1 | | 4 | | | no serious | | serious | | no serious | no serious | | | serious | would not reduce effect | | | no | | no | | very low |
| Zhao 2024 | Assisted reproductive technologies | | with vs without | | Postpartum depression | Postpartum depression risk | | | 12 | | 0 | 12 | | 0 | | | serious | | serious | | no serious | no serious | | | serious | would not reduce effect | | | no | | no | | very low |
| Schoretsanitis 2024 | Postpartum hemorrhage | | with vs without | | Postpartum depression | Postpartum depression risk | | | 10 | | 0 | 5 | | 5 | | | no serious | | serious | | no serious | no serious | | | no serious | would not reduce effect | | | no | | no | | very low |
| Schoretsanitis 2022 | Polycystic ovary syndrome | | with vs without | | Postpartum depression | Postpartum depression risk | | | 6 | | 0 | NA | | NA | | | serious | | serious | | no serious | no serious | | | serious | would not reduce effect | | | no | | no | | very low |
| Qiu 2022 | maternal alcohol | | with vs without | | Postpartum depression | Postpartum depression risk | | | 6 | | 0 | 6 | | 0 | | | no serious | | serious | | no serious | no serious | | | no serious | would not reduce effect | | | no | | no | | very low |
| Neda-Stepan 2024 | neuroticism | | with vs without | | Postpartum depression | Postpartum depression risk | | | 13 | | 0 | 7 | | 6 | | | no serious | | serious | | no serious | no serious | | | serious | would not reduce effect | | | no | | no | | very low |
| Pacho 2023 | substance abuse | | with vs without | | Postpartum depression | Postpartum depression risk | | | 18 | | 0 | NA | | NA | | | no serious | | serious | | no serious | no serious | | | no serious | would not reduce effect | | | yes | | no | | low |
| Lu 2024 | Postpartum pain | | with vs without | | Postpartum depression | Postpartum depression risk | | | 8 | | 0 | NA | | NA | | | no serious | | serious | | no serious | no serious | | | serious | would not reduce effect | | | no | | no | | very low |
| Minaldi 2020 | hyroid autoimmunity | | TPOAb- vs TPOAb+ | | Postpartum depression | Postpartum depression risk | | | 5 | | 0 | 5 | | 0 | | | no serious | | no serious | | no serious | no serious | | | serious | would not reduce effect | | | no | | no | | low |
| Mo 2022 | perinatal pain | | with vs without | | Postpartum depression | Postpartum depression risk | | | 10 | | 0 | 3 | | 2 | | | no serious | | serious | | no serious | no serious | | | no serious | would not reduce effect | | | no | | no | | very low |
| Li 2023b | Neuraxial analgesia during labor | | with vs without | | Postpartum depression | Postpartum depression risk | | | 13 | | 0 | 13 | | 0 | | | no serious | | serious | | no serious | no serious | | | no serious | would not reduce effect | | | no | | no | | very low |
| Fu 2024 | Adverse childhood experiences | | with vs without | | Postpartum depression | Postpartum depression risk | | | 24 | | 0 | 13 | | 11 | | | no serious | | serious | | no serious | no serious | | | serious | would not reduce effect | | | no | | no | | very low |
| Cadman 2024 | NO2 | | with vs without | | Postpartum depression | Postpartum depression risk | | | 12 | | 0 | 12 | | 0 | | | serious | | no serious | | no serious | no serious | | | serious | would not reduce effect | | | no | | no | | very low |
| Cadman 2024 | PM2.5 | | with vs without | | Postpartum depression | Postpartum depression risk | | | 12 | | 0 | 12 | | 0 | | | serious | | no serious | | no serious | no serious | | | serious | would not reduce effect | | | no | | no | | very low |
| Cadman 2024 | PM10 | | with vs without | | Postpartum depression | Postpartum depression risk | | | 9 | | 0 | 9 | | 0 | | | serious | | no serious | | no serious | no serious | | | serious | would not reduce effect | | | no | | no | | very low |
| Cadman 2024 | road traffic noise(>=65dB) | | road traffic noise(>=65dB)vs <65dB | | Postpartum depression | Postpartum depression risk | | | 11 | | 0 | 11 | | 0 | | | serious | | no serious | | no serious | no serious | | | serious | would not reduce effect | | | no | | no | | very low |
| Cadman 2024 | NDVI | | with vs without | | Postpartum depression | Postpartum depression risk | | | 12 | | 0 | 12 | | 0 | | | serious | | no serious | | no serious | no serious | | | serious | would not reduce effect | | | no | | no | | very low |
| Cadman 2024 | Green access | | with vs without | | Postpartum depression | Postpartum depression risk | | | 12 | | 0 | 12 | | 0 | | | serious | | no serious | | no serious | no serious | | | serious | would not reduce effect | | | no | | no | | very low |
| Cadman 2024 | Blue access | | with vs without | | Postpartum depression | Postpartum depression risk | | | 12 | | 0 | 12 | | 0 | | | serious | | no serious | | no serious | no serious | | | serious | would not reduce effect | | | no | | no | | very low |
